# Dual contraceptive utilization and determinant factors among HIV positive women in Ethiopia: a systematic review and meta-analysis, 2020

**DOI:** 10.1186/s40834-021-00161-w

**Published:** 2021-07-01

**Authors:** Alemu Degu Ayele, Bekalu Getnet Kassa, Fentahun Yenealem Beyene, Dagne Addisu Sewyew, Gedefaye Nibret Mihretie

**Affiliations:** 1 Department of Midwifery, College of Health Sciences, Debre Tabor University, Debre Tabor, Ethiopia; 2 Department of Midwifery, College of Medicine and Health Sciences, Bahir Dar University, Bahir Dar, Ethiopia

**Keywords:** Dual contraceptive, Utilization, Determinants, Systematic review, Meta-analysis, Ethiopia

## Abstract

**Background:**

Dual contraceptive is the use of a barrier like condom along with any modern contraceptive methods which has double significance for the prevention STI including HIV and unintended pregnancy. The prevalence and determinants of dual contraceptive utilization described by different studies were highly inconsistent in Ethiopia. Therefore, this systematic review and meta-analysis aimed to estimate the pooled prevalence and determinants of dual contraceptive utilization among HIV positive women in Ethiopia.

**Methods:**

International database mainly Pub Med, Google scholar, HINARI, EMBASE, Cochrane Library, AJOL was applied to identify original studies. STATA software version 14 was applied to analyze the pooled prevalence of dual contraceptive. *I*^2^ test statistics was computed to check the presence of heterogeneity across the studies and eggers test was used to identify publication bias. The pooled prevalence of dual contraceptive utilization was estimated by using a random effects model. The associations between determinants and dual contraceptive utilization were evaluated by using both random and fixed effect models.

**Result:**

A total 9 studies with 9168 HIV positive women were enrolled in this study. The pooled prevalence of dual contraceptive utilization among HIV positive women in Ethiopia was 26.14% (95% CI 21.20–31.08). Disclosure of HIV status (OR = 4.18,95%CI:2.26–7.72), partner involvement in post-test counselling (OR = 2.31,95%CI:1.63–3.25), open discussion about dual contraceptive with partner (OR = 4.27 95% CI:1.69–10.77), provision of counselling on dual contraceptives by health care provider (OR = 4.47,95% CI:3.81–5.24) and CD4 count > 350 cells/ mm^3^ (OR = 3.87,95%CI:3.53–4.23) were among the significant factors associated with dual contraceptive utilization.

**Conclusion:**

The overall prevalence of dual contraceptive utilization among HIV positive women was significantly low. Disclosure of HIV status, partner involvement in post-test counselling, open discussion about dual contraceptive with partner, counselling on dual contraceptive by health care provider and CD4 count > 350 cells/ mm^3^ were positively affect dual contraceptive utilization. This study implies the need to develop plans and policies to improve partner involvement posttest counseling, integrate the counseling and provision of dual contraceptive at ART clinic at each level of health system.

**Supplementary Information:**

The online version contains supplementary material available at 10.1186/s40834-021-00161-w.

## Background

Human immune deficiency virus is the most common public health issue and continues to have disastrous multidimensional impacts on individuals, females, nations and the international community at large [1]. UNAIDS 2019 report, indicated that in the globe 37.9 million peoples were living with HIV, 1.7 million people became newly infected with HIV and around 770,000 people died from AIDS-related illnesses. In addition, an estimated number of 600 young women aged from 15 to 24 become infected by HIV. Among these new infections four out of five were from the sub-Saharan Africa [2]. This showed that the burden is highest in Sub Saharan Africa countries where, 60% of people reported to live with HIV/AIDS and more than half of them were females [3]. Likewise, Ethiopia is among the countries mostly affected by the HIV epidemic. According to the Ethiopian Demographic Health Survey (EDHS) 2016 report, the national adult HIV prevalence was 0.9% with women disproportionately infected, 1.2% compared to 0.6% in men [4].

Worldwide, due to poor contraceptive utilization and unsafe sex practices, greater than 2 million HIV positive women become pregnant every year, out of these, 600,000 die due to pregnancy related complications [5]. Besides, unintended pregnancies responsible for 21.3% of new born HIV infections, 90% were from Sub Saharan African countries [6]. Ethiopia is among the sub-Saharan countries which is seriously affected by the epidemic with an estimated number of 24,000 HIV-positive pregnant women and 3800 new HIV infections among children in the 2016 [7].

HIV positive women can use most of the available contraceptive methods to limit and/or space their fertility including, hormonals, intra uterine device and sterilization. However, these methods do not protect against the transmission of both STIs/HIV between partners. At the same time, the efficacy of estrogen based hormonal contraceptive is also affected by drug to drug interaction with some Antiretroviral (ARV) drugs [8–10]. Due to these and other reasons HIV positive women needs barrier methods like condom in addition to the method the used to prevent unintended pregnancy.

Utilization of dual contraceptive is the use of two different methods, a barrier like condom along with any modern contraceptive methods which has double significance for those HIV positive women by preventing transmissions of STI including HIV and unintended pregnancy [11, 12].

In Ethiopia context different primary studies were conducted to estimate the prevalence of dual contraceptive utilization among HIV positive women [13–30] (Tewabe T, Abdanur A, Jenbere D, Ayehu M, Talema G: Contraceptive use and associated factors among sexually active HIV positive women attending ART clinic in FHRH in Bahir Dar, north west, Ethiopia. Facility based cross-sectional study. 2018. BMC Contraceptive and Reproductive Medicine Research Square Preprint current status, under review). These detached studies showed that prevalence of dual contraceptive utilization among HIV positive women in Ethiopia ranges from13.2% in University of Gondor Hospital Northwest Ethiopia [14] to 59.5 in West zone health facilities, Oromia Ethiopia [21]. From the reports of these studies there was a great variation and inconsistency related to prevalence of dual contraceptive utilization among HIV positive women throughout the country.

Women age, disclosure of HIV status to partner, partner involvement in post-test counselling, open discussion with partner on dual contraceptive use, provision of counseling on dual contraceptive by health care providers, having HIV negative partner and CD4 count were most frequently (twice and/or more) reported determinants of dual contraceptive utilization among HIV positive women in Ethiopia [13, 14, 18, 21, 24, 25, 28–30].

The reasons for the above variation in the prevalence and determinants of dual contraceptive utilization among Ethiopian HIV positive women’s have not yet been investigated. Hence, it is important to have summarized evidence on the prevalence rate and determinant factors of dual contraceptive utilization among HIV positive women, as this helps the concerned bodies to identify existing gaps and propose supplementary strategies to increase the availability, accessibility and utilization of dual contraceptive in Ethiopia. Therefore, the aim of this study was to summarize the evidence of dual contraceptive utilization and determinant factors among HIV positive women in Ethiopia.

## Methods

### Study design and protocol

This study was a systemic review and meta-analysis of both published and unpublished relevant articles. The result of this meta-analysis was reported via systematic review and meta-analyses reporting PRISMA guidelines [31]. Protocol registration is not applicable in this systematic review and meta-analysis.

### Eligibility criteria

#### Inclusion criteria

##### Study area

Only studies conducted in Ethiopia were included.

##### Publication condition and period

Both published and unpublished articles from August 01 /2010 to August 01/ 2020 were considered.

##### Study design

Cross-sectional studies which contain original data reporting the prevalence or/and determinants of dual contraceptive utilization was considered.

##### Population

Studies on HIV positive women were considered.

#### Exclusion criteria

Studies without full content, anonymous reports, editorials, and qualitative studies were excluded. In addition, we exclude articles which were not fully accessed after we make contact with the primary author two times through email.

### Searching strategy and data source/data base

Major international databases such as PubMed, HINARI, EMBASE, Cochrane Library, Google Scholar, and African Journals Online databases were used to search relevant published studies. Google hand searching was also performed for unpublished studies.

The PECO (Population, Exposure, Comparison and Outcomes) search format has used this review to search pertinent studies.

#### Population

HIV positive women.

#### Exposure

Determinants of dual contraceptive utilization (socio-demographics such as age, educational status,) reproductive related factors (number of children, desire of future fertility, partner involvement in post -test counselling), health care and disease related factor (having HIV negative partner, disclosure of HIV status to partner, counseled about FP by health care provider, open discussion on dual contraceptive with partner).

#### Comparison

The reported reference groups for each determinant factor in each respective study such as, dual contraceptive utilization among HIV positive women who have open discussion with partner versus those who haven’t open discussion, and dual contraceptive utilization among HIV positive women whose husband has involved in the post-test counselling versus their counterparts.

#### Outcome

Dual contraceptive utilization among HIV positive women.

Studies were searched exhaustively by 3 authors (AD, BG and FY) from international databases by using comprehensive searching strategies. Initially, pretested search strategies were developed to retrieve all pertinent studies. First, articles were searched by examining the full titles (“Dual contraceptive utilization and determinant factors among HIV positive women in Ethiopia”) and then keywords (“dual contraceptive”, “family planning”,” “prevalence”, magnitude, “determinants”, “predictors”, “associated factors”, HIV positive”, “reproductive age”, “group,” “women”, and “Ethiopia”). These keywords were used independently and in combination using Boolean operators “OR” or “AND”. Besides to this, studies were also searched from the reference lists of all included studies to find any other missed studies by our searching strategies. Furthermore, to find pertinent unpublished studies, Ethiopian universities digital libraries were searched (Addis Ababa University, University of Gondar and Haramiya University). The searching periods were from September 15, 2020 to October 15, 2020. Finally, all studies identified by our search strategy were imported and managed using Endnote X7 reference manager software (Additional file 1).

#### Identification and study selection

In the screening phase, three independent authors (AD, BG, FY) assessed the articles independently for inclusion and exclusion through a title, abstract and full review by using the search strategy. Any divergences and disagreement between three reviewers were managed by a discussion and consensus based on established criteria and if necessary, with the fourth and fifth authors (DA and GN). In the second phase of screening, those potentially eligible studies were undergoing full-text review to determine if they satisfy the predetermined inclusion criteria and assessed for duplicated records. When duplicate data were encountered, only the full-text article published was retained.

### Quality assessment

The scientific strength and quality of each incorporated original cross-sectional study was assessed by using the Newcastle-Ottawa Scale quality assessment tool adapted for cross-sectional study quality assessments [32]. The tool has three core components; the principal component of the tool graded from five stars and mainly emphasized on the methodological quality of each primary study. The second component of the tool graded from two stars and mainly concerns about the comparability of each study and the last component of the tool graded from three stars and used to assess the outcomes and statistical analysis of each original study. The quality of each original study was assessed by three authors independently using this tool. Disagreements between the three authors were resolved by the last two authors. Finally, the quality of the studies was weighed up based on these components. Overall, the quality of the eligible studies was moderate (Additional file 2).

#### Data extraction

All necessary data were extracted from 19 primary studies by three reviewers (AD, BG & FY) individually using pre tested standardized data extraction form. This form includes primary author, year of publication, study setting, sample size, study design, response rate, statistically significant factors, adjusted Odds ratio (AOR), 95% confidence interval, and covariance is considered. Uncertainties during the extraction process were resolved by logical consensus between the three authors and the final consensus was approved by the last two authors (DA and GN). In the case of incomplete data, we excluded the study or the parameter that was not available.

For the second objectives (determinants), the information extraction format was prepared for each specific determinant, i.e., women age, HIV status disclosure to partner, partner involvement in post-test counseling, open discussion with partner on dual contraceptive use, provision of counseling on dual contraceptive by health care providers, having HIV negative partner and CD4 count. In this study, variables were selected if at least two or more studies reported them as a significant factor.

### Outcome of interest

The primary outcome of this systematic and meta-analysis was the prevalence of dual contraceptive utilization among HIV positive Ethiopian women. The second objective of the review was to find out the determinants of dual contraceptive utilization.

### Publication bias and heterogeneity

To minimize the risk of bias comprehensive searches (electronic/database search and manual search) were used. Cooperative work of the authors was also critical in reducing bias, in selection of articles based on the clear objectives and eligibility criteria, deciding the quality of the article, in regularly evaluating the review process, and in extracting and compiling the data.

We examine publication bias with visual inspection of the funnel plot graph qualitatively. Besides, Egger’s correlation tests at a 5% significant level were done to assess the presence of publication bias [33, 34]. Furthermore, to reduce the random variations among the point estimates of the primary study, subgroup analysis was done based on the study region, publication year and sample size. Heterogeneity across studies was evaluated using invers variance (I^2^) statistics with its corresponding *p*-value using random effect model.

### Statistical analysis

To provide a comparable classification of the outcome and determinants of interest a Meta-analysis was performed for selected articles. The associated factors of dual contraceptive utilization were examined based on eligibility criteria. We had considered at least two studies were reported about one associated factors of dual contraceptive in common with their measure of effect and 95% confidence interval (CI). The significant association between factors affecting dual contraceptive utilization was estimated by calculating the effect size and 95% confidence interval (CI). Random effects model based on DerSimonian-Laird method was considered to assess for variations between the studies as well as within the studies. Data abstraction and analysis were done by using Microsoft Excel and STATA software version 14 respectively. The results were presented using texts, tables and forest plots with measures of effect and 95% confidence interval. Statistical heterogeneity was tested via the I^2^ statistics at a *p* value ≤0.05 [35].

## Results

### Description of studies

**Fig. 1:**
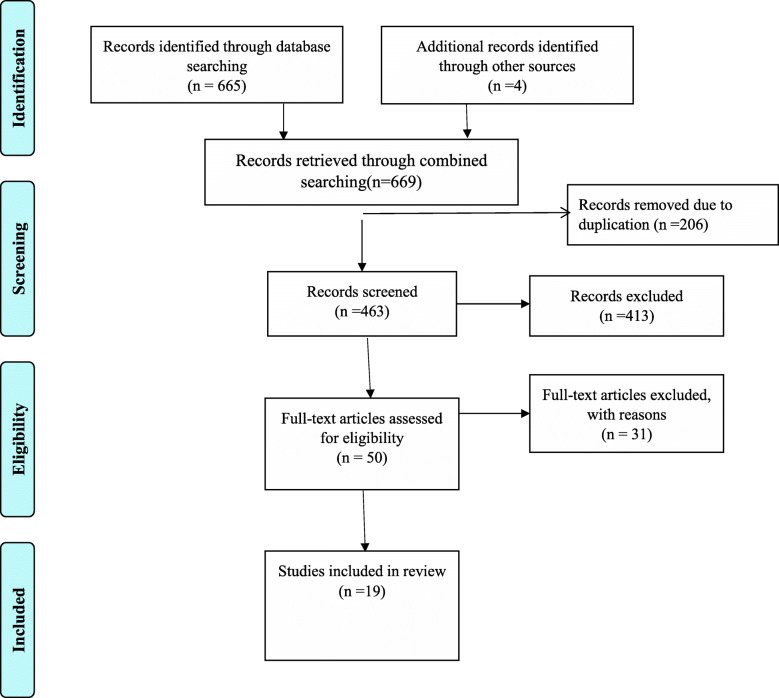
Flow chart describing the selection of studies for the systematic review and meta-analysis of prevalence and determinants of modern contraceptive utilization among HIV positive women’s in Ethiopia, 2020

Our literature search identified 669 primary studies by using medical electronic databases and other relevant sources searching mechanisms. Among the identified studies, 206 articles were removed after reviewing their titles due to duplication whereas 463 articles were allowed for further screening. Out of these 413 articles were removed due to irrelevance after being screened based on titles and abstracts. Then after the remaining 50 articles were assessed for eligibility and 31 of them were excluded due to inappropriate use of statistical analysis, irrelevant target population, inconsistent study report, outcome of interest not reported, unavailability of full text and inconsistency with the predetermined inclusion criteria for the review. Finally, 19 articles were included in the systematic review and meta-analysis (Fig. 1).

### Characteristics of the included studies

All the 19 studies eligible for the current systematic review and meta-analysis were cross-sectional by study design, reported with English and conducted from 2013 to 220. The sample size ranges from 243 in Gebretsadik Shawo Hospital, found in South Nation Nationalities and peoples representatives (SNNPR), South West Ethiopia [30] to 1418 in Addis Ababa [17]. In this systematic review and meta-analysis, a total of 9168 HIV positive women were enrolled to estimate the pooled prevalence of dual contraceptive use. Regarding to geographical distribution of studies, six studies were from Oromia [18–23], five studies were from Amhara [13–16] (Tewabe T, Abdanur A, Jenbere D, Ayehu M, Talema G: Contraceptive use and associated factors among sexually active HIV positive women attending ART clinic in FHRH in Bahir Dar, north west, Ethiopia. Facility based cross-sectional study. 2018. BMC Contraceptive and Reproductive Medicine Research Square Preprint current status, under review), four studies from Tigray [24–27], two from SNNP [29, 30], one study from Addis Ababa [17] and one from Benishangul Gumuze [28]. The highest and lowest prevalence of dual contraceptive utilization were 59.5 and 13.2% respectively. In addition, the response rate of the primary studies incorporated in the current study range from 93.2 to 100%. Among the 19 included studies 17 of them were published articles in reputable journal while two of them [25] (Tewabe T, Abdanur A, Jenbere D, Ayehu M, Talema G: Contraceptive use and associated factors among sexually active HIV positive women attending ART clinic in FHRH in Bahir Dar, north west, Ethiopia. Facility based cross-sectional study. 2018. BMC Contraceptive and Reproductive Medicine Research Square Preprint current status, under review) were repository articles (Table 1). Lastly, the primary studies included in the present systematic review and meta-analysis had a quality score of 6–9 out of 10 points (Additional file 2).

**Table 1:** Characteristics of included primary studies showing dual contraceptive utilization and determinants among HIV positive women in Ethiopia, systematic review and meta-analysis, 2020

Authors	Year of publication	Region	Study design	Sample size	Response rate (%)	Prevalence (95%CI)	Quality
Abay et al. [13]	2020	Amhara	C/S	563	98	28.8 (26.83,30.77)	Low risk
Reta et al. [14]	2019	Amhara	C/S	619	98.25	13.2 (11.72,14.68)	Low risk
Kebede et al. [15]	2015	Amhara	C/S	420	99.04	26.7 (24.77,28.63)	Low risk
Egzeabher et al. [16]	2015	Amhara	C/S	335	98.5	19 (17.29,20.71)	Low risk
Tewabe et al.	2018	Amhara	C/S	308	100	26 (24.09,27.91)	Low risk
Asfaw et al. [17]	2014	AA	C/S	1418	100	18 (16.32,19.68)	Low risk
Demissie et al. [18]	2015	Oromia	C/S	357	100	32 (29.96,34.04)	Low risk
Sufa et al. [19]	2013	Oromia	C/S	461	98.9	22.4 (20.58,24.22)	Low risk
Polis et al. [20]	2014	Oromia	C/S	395	93.2	17 (15.36,18.64)	Low risk
Dereje et al. [21]	2019	Oromia	C/S	323	94.1	59.5 (57.36,61.64)	Low risk
Feyissa et al. [22]	2020	Oromia	C/S	360	100	17 (15.36,18.64)	Low risk
Tesfaye et al. [23]	2014	Oromia	C/S	403	99.5	31.9 (29.86,33.94)	Low risk
Gebrehiwot et al [24]	2017	Tigray	C/S	331	97.9	15.7 (14.11,17.29)	Low risk
Kalayu [25]	2019	Tigray	C/S	632	100	45.2 (43.03,47.37)	Low risk
Melaku et al. [26]	2014	Tigray	C/S	964	100	14 (12.48,15.52)	Low risk
Berhane.et al. [27]	2013	Tigray	C/S	364	99.2	21.6 (19.80,23.40)	Low risk
Assefa et al. [28]	2014	Benishangul	C/S	404	100	40.9 (38.75,43.06)	Low risk
Markos et al. [29]	2019	SNNP	C/S	269	95.9	28.3 (26.34,30.26)	Low risk
Meseret et al. [30]	2015	SNNP	C/S	243	98.78	19.8 (18.06,21.54)	Low risk

*AA* Addis Ababa, *SNNP* Southern Nations Nationalities Peoples, *C/S* Cross-sectional

### Dual contraceptive utilization

**Fig. 2:**
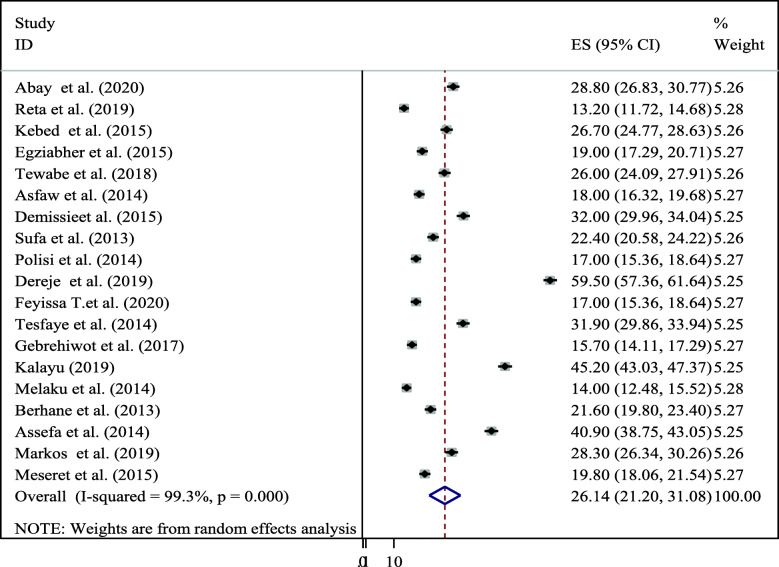
Forest plot of the pooled prevalence of dual contraceptive use among HIV positive women in Ethiopia, 2020

The overall pooled prevalence of dual contraceptive utilization among HIV positive women in Ethiopia was 26.14% (95% CI 21.20–31.08) (Fig. 2).

### Heterogeneity and publication bias

According to the finding of this study there was a markedly high heterogeneity across the studies as evidenced by I^2^ statistics (I^2^ = 99.3%, *P* ≤ 0.001). Due to this random effect model meta-analysis was applicable to determine the pooled prevalence of dual contraceptive utilization among HIV positive women in Ethiopia.

**Fig. 3:**
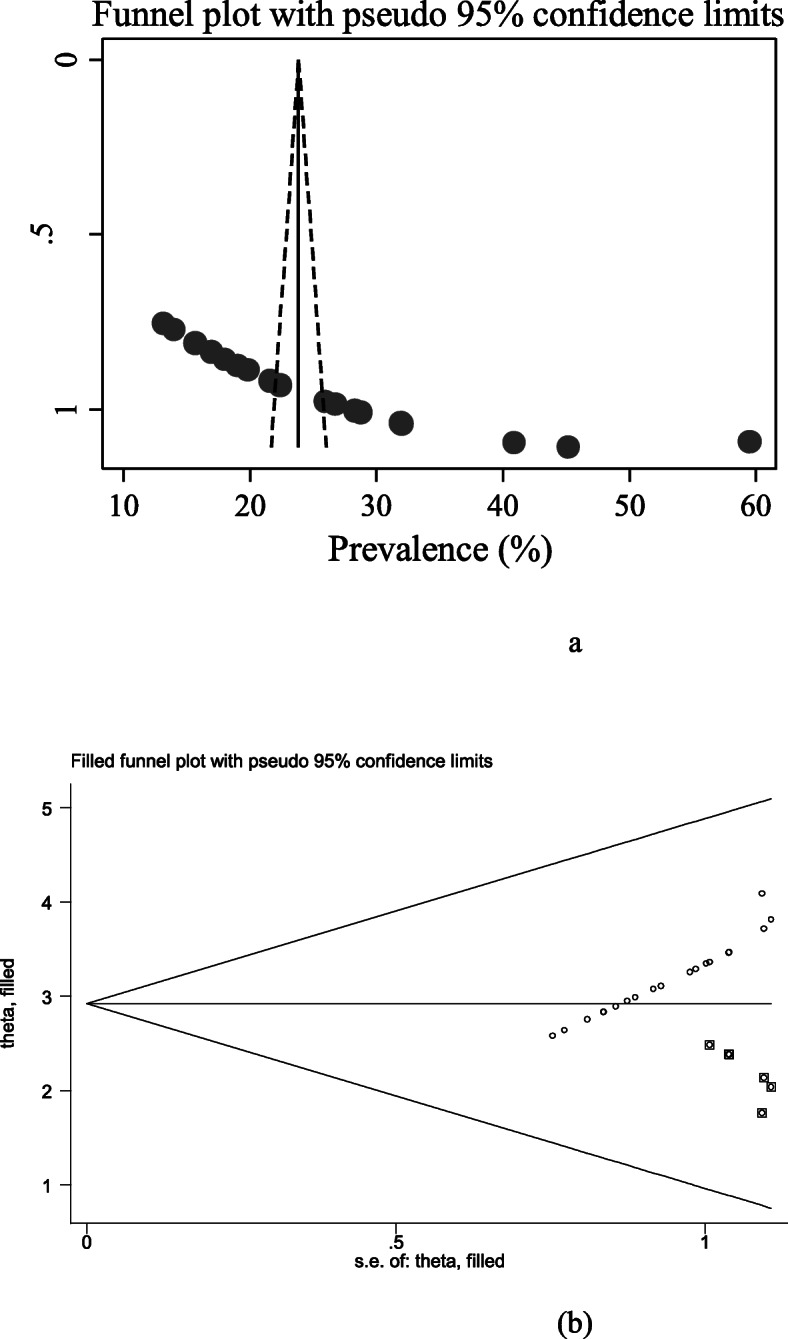
**a** Funnel plot to test publication bias of 19 studies. **b** Result of trim and fill analysis for adjusting publication bias of the 25 studies

Publication biases among the included studies were examined by using both funnel plots and egger’s regression test. The results of funnel plots showed an asymmetric shape, which indicates the presence of publication bias among those included studies (Fig. 3a). Objective assessments of publication bias by egger’s regression test also showed the presence of publication bias across studies (*p*-value < 0.001). The Duval and Tweedie nonparametric trim and fill analysis were done to correct publication bias among the studies. Accordingly, publication bias was corrected when six missed studies were filled in the funnel plot by trim and fill analysis After six studies were filled, a total of 25 studies were included and computed via the trim and fill analysis to produce the pooled prevalence of 19.519% (95% CI, 13.744–25.294) by applying random effect model (Fig. 3b).

### Sensitivity analysis

In the current meta-analysis, in order to determine the potential source of heterogeneity seen in the pooled prevalence of dual contraceptive utilization, the investigators performed a leave-one-out sensitivity analysis. The result of the sensitivity analysis indicated that the finding was not relay on a particular study. The pooled prevalence of dual contraceptive utilization was varied and ranges from 24.28% (20.37–28.18%) to 26.86% (21.79–31.92%) after deletion of six studies (Table 2).

**Table 2:** Sensitivity analysis of the prevalence of dual contraceptive utilization among HIV positive women in Ethiopia 2020

Study omitted	Prevalence (%)	95%CI
Abay et al. (2020) [13]	25.99	20.82–31.16
Reta et al. (2019) [14]	26.86	21.79–31.95
Kebede et al. (2015) [15]	26.11	20.92–31.30
Egzeabher et al. (2015) [16]	26.54	21.34–31.74
Tewabe et al. (2018)	26.15	20.95–31.35
Asfaw et al. (2014) [17]	26.59	21.40–31.78
Demissie et al. (2015) [18]	25.81	20.69–30.93
Sufa et al. (2013) [19]	26.35	21.13–31.56
Polis et al. (2014) [20]	26.65	21.47–31.82
Dereje (2019) [21]	24.28	20.37–28.18
Feyissa et al. (2020) [22]	26.65	21.47–31.82
Tesfaye et al. (2014) [23]	25.82	20.69–30.94
Gebrehiwot et al. (2017) [24]	26.72	21.57–31.87
Kalayu (2019) [25]	25.08	20.31–29.85
Melaku et al. (2014) [26]	26.81	21.72–31.91
Berhane et al. (2013) [27]	26.39	21.17–31.60
Assefa et al. (2014) [28]	25.32	20.40–30.23
Markos et al. (2019) [29]	26.02	20.84–31.19
Meseret et al. (2015) [30]	26.49	21.28–31.70

### Subgroup analysis

We performed a subgroup analysis based on the region where the primary studies were done. As a result, the largest prevalence was observed in Oromia with a prevalence of 29.96% (95%CI, 17.98–41.93) and lowest in Amhara with prevalence of 22.72% (95%CI, 16.66–28.78). In addition, subgroup analysis was also checked by publication year and those studies published from 2019 to 2020 has the highest prevalence, 31.99(18.31–45.67) while, studies published from 2016 to 2018 has the lowest pooled prevalence 20.84(10.74–30.93). Lastly, we also executed subgroup analysis using sample size (< 400 and > 400) of the primary studies. The outcome of this subgroup analysis revealed that studies done with sample size of greater than 400 26.77% (95% CI, 19.60–33.94) were slightly higher in utilization of dual contraceptive compared to those studies done with sample size of greater than 400, 25.58(18.38–32.77) (Table 3).

**Table 3:** Subgroup analysis of dual contraceptive utilization among HIV positive women in Ethiopia

Variables	Characteristics	Included studies	Number of study participants	Prevalence (95% CI)	I^2^(%), *P-value*
Region	Amhara	5	2245	22.72 (16.66–28.78)	98.3, < 0.001
	Oromia	6	2299	29.96 (17.98–41.93)	99.6, < 0.001
	A. A	1	1418	18.00 (16.32–19.63)	–
	Tigray	4	2291	24.11 (11.79–36.42)	99.5, < 0.001
	Benishangul	1	403	40.90 (38.75–43.05)	–
	SNNP	2	512	24.04 (15.71–32.37)	97.5, < 0.001
Sample size	≤400	10	3285	25.58 (18.38–32.77)	99.4, < 0.001
	> 400	9	,5883	26.77 (19.60–33.94)	99.3, < 0.001
Year of publication	2013–2015	11	5763	23.91 (19.43–28.40)	98.5, < 0.001
	2016–2018	2	639	20.84 (10.74–30.93)	98.5, < 0.001
	2019–2020	6	2766	31.99 (18.31–45.67)	99.7, < 0.001
Overall		19	9168	26.14 (21.20–31.08)	99.3, < 0.001

### Determinants of dual contraceptive utilization

The finding of this systematic review and meta-analysis revealed that dual contraceptive utilization among HIV positive women in Ethiopia were significantly associated with disclosure of HIV status to partner, partner involvement in post-test counselling, open discussion about dual contraceptive with partner, provision of counseling on dual contraceptive by health care providers, CD4 count >350cells/dl whereas having HIV negative partner is mentioned as a determinant factor for dual contraceptive use.

**Fig. 4:**
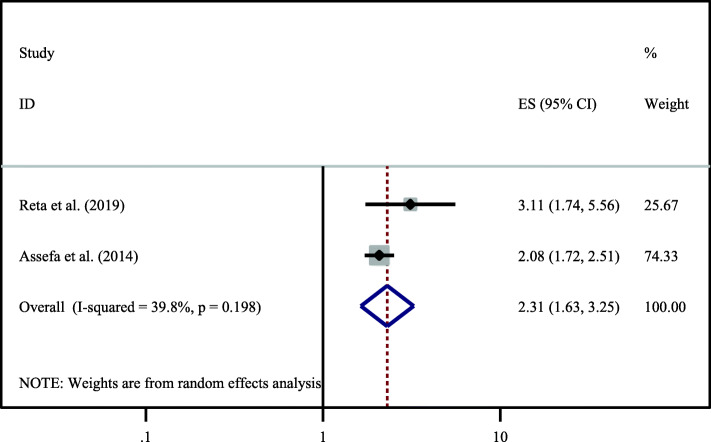
Association between partner involvment in post-test counseling with dual contraceptive utilization

According to this study two primary articles identified that partner involvement in post-test counselling was significantly associated with dual contraceptive utilization [14, 28]. Those women whose partner was involved in the post-test counseling were 2.31 times (OR = 2.31,95%CI:1.63, 3.25) more likely to use dual contraceptive than those women whose husband was not participated in the post-test counselling. Heterogeneity test indicated I^2^ = 39.8%, hence random effect model was applicable for analysis (Fig. 4).

**Fig. 5:**
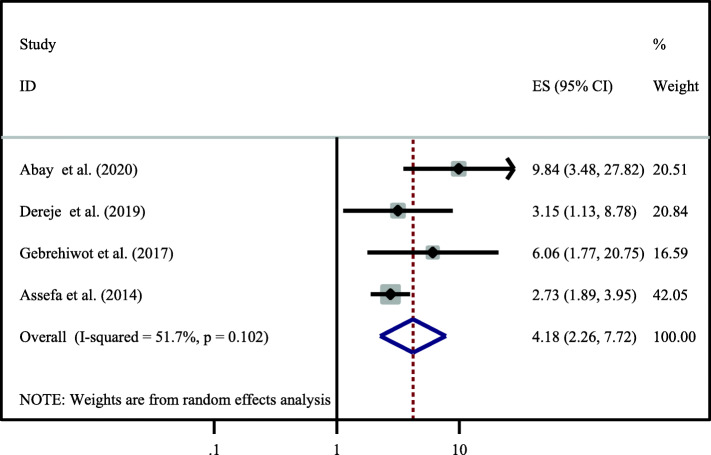
Association between disclosure HIV status to partner with dual contraceptive utilization. Moreover, six primary studies also reported that open discussion on the issue dual contraceptive

Based on the finding of the current study four primary studies showed that disclosure of HIV status to partner were significantly associated with dual contraceptive utilization [13, 21, 24, 28]. The odds of utilizing dual contraceptive methods were four times (OR = 4.18,95%CI:2.26–7.72) higher among those who disclose their HIV status to their husband as compared to their counterparts. Since the heterogeneity test showed I^2^ = 51.7% we used random effect model for analysis (Fig. 5).

**Fig. 6:**
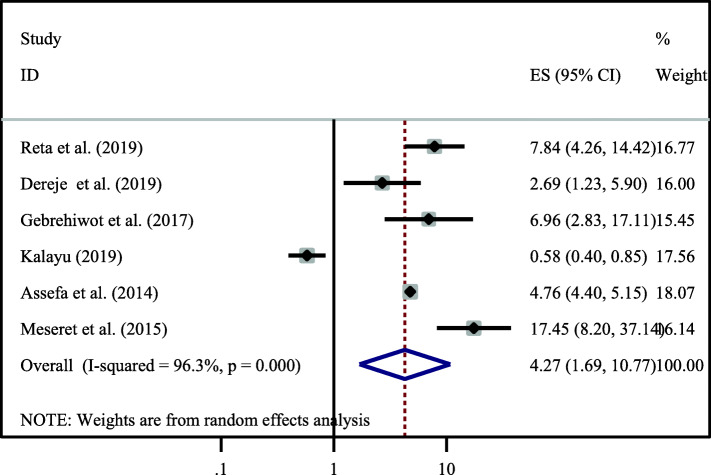
Association between open discussion about dual contraceptive with partner and utilization of it

Moreover, six primary studies also reported that open discussion on the issue dual contraceptive with partner was strongly associated with dual contraceptive use [14, 21, 24, 25, 28, 30]. HIV positive women who has open discussion on the issue of dual contraceptive use with their partner were 4.27 times (OR = 4.27, 95% CI:1.69–10.77) higher the odds of utilizing dual contraceptive as compared to those women who has no open discussion. Marked heterogeneity was observed across the studies (I -squared = 96.3%). As a result, random effect model analysis was used to assess the association between two variables (Fig. 6).

**Fig. 7:**
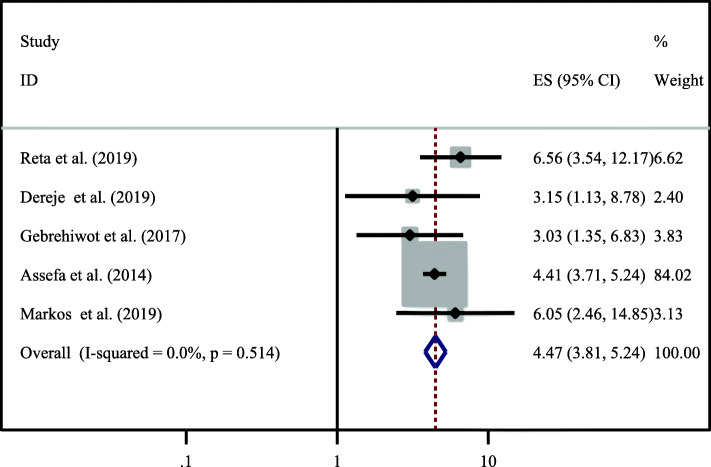
Association between provision of counseling on dual contraceptive by health care providers and utilization of it

The result of this systematic review and meta-analysis concluded that getting counseling about dual contraceptive use by health care provider was another significant predictor of dual contraceptive use as mentioned by five primary studies [14, 21, 24, 28, 29]. The likely hood of using dual contraceptive methods in HIV positive women who has got counseling on dual method use by health care provider was 4.47 times (OR = 4.47,95% CI:3.81–5.24) higher than their counterparts. The heterogeneity test showed an I value of 0.0%, hence, we used fixed effect model for analysis (Fig. 7).

**Fig. 8:**
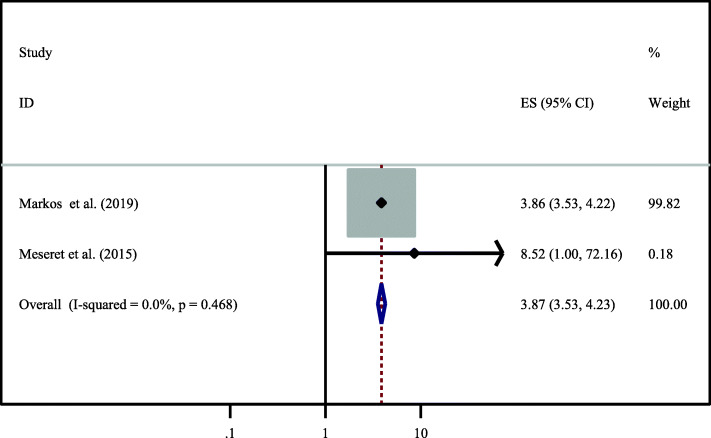
Association between CD4 count with dual contraceptive utilization

Finally, the association between dual contraceptive utilization and CD4 count was evaluated by using 2 studies [28, 30]. Dual contraceptive utilization was significantly associated with CD4 count, mentioned in two primary studies. HIV positive women who has CD4 count of > 350 cells/ mm^3^ were almost four times (OR = 3.87,95%CI:3.53–4.23) higher the odds of utilizing dual contraceptive methods as compared to women who has CD4 count of < 250. The investigators used a fixed effect model for the analysis because the I^2^value was 0.0% (Fig. 8).

## Discussion

The hall mark of utilizing dual contraceptive methods includes the prevention of both unintended pregnancy and HIV transmission. It also has an added benefit in reducing the number of unsafe abortion and getting HIV free generation and as large improving the health status of the community. Hence, the current systematic review and meta-analysis is aimed to estimate the pooled prevalence of dual contraceptive utilization and its determinants among HIV positive women in Ethiopia. According to this meta-analysis, the pooled prevalence of dual contraceptive utilization among HIV positive women in Ethiopia was 26.14% (95% CI:21.20–31.08). This indicates that almost one from four women has been utilize dual contraceptive methods. The finding of this study was in lined with a multinational study done on African women (23.5%) [36], Sub-Saharan Africa (31%) [37], India (29.9%) [38], Malawi (26.5%) [39]. On the other hand, the finding of this study is lower than a survey conducted in USA (39%) [12]. This might be due to socio-demographic differences. Furthermore, the finding is lower than a survey done in Cameron (33.3%) [40], South Africa (33%) [41], Kenya (38.5%) [42]. This discrepancy might be due to the differences in women’s negotiation ability to use condom as additional contraceptives due to socio cultural impact, respondents’ level of knowledge about unique benefit of using dual contraceptive methods other than preventing unintended pregnancy, availability of dual contraceptives and level of partner involvement between Ethiopia and across the respective countries.

However, the current pooled prevalence is higher than a survey performed in Zambia (17.7%) [43], Togo16.9% [44] South Malawi (18.9%) [45]. The possible explanation might be partly as a result of difference in socio-cultural parameters, study setting, time gaps of the primary studies conducted in the comparable countries. Nowadays the government of Ethiopia is highly committed in expanding of family planning programs including condom distribution that might be positively affect dual contraceptive.

In the present study we found significant dual contraceptive utilization among HIV positive women whose partner was involved in post-test counselling. Women whose partners were involved in post-test counselling were more likely to utilize dual contraceptives as compared to their counterparts. The finding is in agreement with study done in India [38]. This may be the reflection of involving partner in testing and counselling process help both of them to have open discussion in selecting the preferred contraceptive they utilize. The other reason might be now a day in Ethiopia, male partner involvement in the prevention of mother to child transmission of HIV/AIDS and related reproductive health services become highly advocated and exercised to scale up dual contraceptive utilization which eventually reduce the burden of both unintended pregnancy and HIV/AIDS.

In addition, women who have open discussion with partner regarding to dual contraceptive utilization increase the odds of utilizing dual contraceptives as compared to women who have not open discussed with their partner. This is in agreement with study conducted in India [46], south Africa [47], Tanzania [48], Northern Uganda [49]. Open discussion with partner may empower women to make informed decision on fertility and contraceptive options, inspire to disclose their HIV status and general speaking it play an important role to manage life very simply. In addition, open discussion may increase women’s freedom to negotiate male partner in decision of limiting or spacing number of births and safer sex practice. Furthermore, in the majority of our community male partner plays a great role in decision making role either to support or prohibit the use of dual contraceptive thus open discussion is crucial to minimize/eliminate challenges encounter in using condom consistently and correctly in each sexual activity.

According to the finding of current meta-analysis, disclosing of HIV status to male partner is another crucial to scale up utilization of dual contraceptives. This is consistent with a study conducted in Kenya [42], Uganda [50], Lusaka Zambia [51]. This might be due to the fact that stigma/discrimination and misconception-related HIV/AIDS was reduced due to community awareness and higher partner care which leads HIV positive women to disclose their status to their partner. Moreover, HIV status disclosure is imperative to get support and facilitates open discussion with male partner in regard to safer sex practice. Disclosing HIV status also help the women to have common understanding regarding to the importance of dual contraceptives utilization and help to reach an agreement to utilize it.

Moreover, provision of counseling on dual contraceptive utilization by health care providers were significantly associated with utilization of them. Our finding is consistent with a study finding from Kenya [42], Tanzania [48], Zambia [43], Northern Uganda [49]. This might be due to provision of counseling empowers and allows women to make informed decision which may increase the uptake of dual methods. Counseling also offers information regarding to the importance of safer sex, build self-efficacy, and negotiation skills on consistent and correct use of condoms. On the other hand, counseling and providing of reproductive health interventions may have a positive influence on minimizing unmet need for dual contraceptive utilization.

Once more, the current meta-analysis finding showed that women who had CD4 count > 350 cells/ mm^3^ were more likely to use dual methods than women with CD4 count < 250 cells/ mm^3^. This was supported by study done in Zambia [43]. On the contrary this finding was inconsistent from study finding from India [38]. Perhaps this might be those women who have higher number of CD4 count may either have children or no farther intention for future fertility which might increase the use of dual methods.

### Limitation

This systematic review and meta-analysis have some limitations. All of the studies included in this systematic review and meta-analysis were cross-sectional in nature; as a result, the outcome variable might be affected by other confounding variables. Beside to this, some of the studies enrolled in the in this systematic review and meta-analysis had small sample size, which may affect the actual magnitude of utilization at the country level. Furthermore, all regions in Ethiopia were not represented in this systematic review and meta-analysis due to limited number of studies in the country (only five regions and one administrative town were represented in this study). As a result, the finding of this systematic review and meta-analysis may not exactly indicate the national prevalence of dual contraceptive utilization.

## Conclusion

The overall prevalence of dual contraceptive utilization among HIV positive women was significantly low in Ethiopia. Male partner involvement in post-test counselling, disclosure of HIV status to partner, open discussion about dual contraceptive with partner, provision of counselling on dual contraceptive and CD4 counts were found to be predictors of dual contraceptive. This study implies the need to develop plans and policies to improve male partner involvement during post-test counseling and provision of counseling on dual contraceptive at each level of health system. Besides to this, improving disclosure of HIV status to male partner and open discussion about dual contraceptive with partner were recommended.

## Supplementary Information

**Additional file 1.** Searching strategy.

**Additional file 2.** Quality assessment.

## Data Availability

The data that support the review findings of this study are available upon a reasonable request to the corresponding author.

## Abbreviations

AIDIS: Acquired Immune Deficiency Syndromes
HIV: Human Immune Virus
FP: Family Planning
WHO: World Health Organization
