# Supplementary material for: Dual contraceptive utilization and determinant factors among HIV positive women in Ethiopia: a systematic review and meta-analysis, 2020
Source: Contracept Reprod Med. 2021 Jul 1;6:19. doi: 10.1186/s40834-021-00161-w (PMC8247245; doi:10.1186/s40834-021-00161-w)
Supplement: Supplementary file 1 — Additional file 1. Searching strategy. [file 40834_2021_161_MOESM1_ESM.docx]

**Additional 1:** Searching strategy for Dual contraceptive utilization and determinant factors among HIV positive women in Ethiopia: a systematic review and meta-analysis,2020.

| Databases | Searching terms | Number of studies |
| --- | --- | --- |
| PubMed | "Dual contraceptive use"[All Fields] OR "contraceptive uptake"[MeSH Terms] OR "contraceptive devices"[All Fields] OR "contraceptive"[All Fields] OR "contraceptive agents"[MeSH Terms] OR "contraceptive agents"[All Fields]) AND associated[All Fields] AND factors[All Fields] OR "associated factors"[MeSH Terms] AND ("HIV seropositivity"[MeSH Terms] OR ("HIV /AIDS"[All Fields] AND "seropositivity"[All Fields]) OR "HIV seropositivity"[All Fields] OR ("HIV"[All Fields] AND "among women living with AIDS"[All Fields]) OR "HIV positive"[All Fields]) AND ("women"[MeSH Terms] OR "women"[All Fields]) AND "Ethiopia"[All Fields]) | 429 |
| Google scholar | “Dual contraceptive use” OR “utilization of family planning,” AND “associated factors” AND “among HIV positive women AND “Ethiopia” | 186 |
| HINARI | “Dual contraceptive use” OR “uptake of contraceptive” OR “utilization of family planning,” OR “modern contraceptive use” OR “uptake of family planning” AND “associated factors” AND “among HIV positive women AND “Ethiopia” | 12 |
| Cochrane library | “Dual contraceptive use” or “modern contraceptive uptake” or “family planning utilization” and “determinants” or “predictors” and “HIV positive women” and “Ethiopia” | 9 |
| EMBASE | “Dual contraceptive use” OR “uptake of contraceptive” OR “utilization of family planning,” OR “modern contraceptive use” AND “associated factors” AND “among HIV positive women AND “Ethiopia” | 22 |
| Others databases |  | 11 |
| Total retrieved |  | 669 |
| Included |  | 19 |
