# Supplementary material for: Dual contraceptive utilization and determinant factors among HIV positive women in Ethiopia: a systematic review and meta-analysis, 2020
Source: Contracept Reprod Med. 2021 Jul 1;6:19. doi: 10.1186/s40834-021-00161-w (PMC8247245; doi:10.1186/s40834-021-00161-w)
Supplement: Supplementary file 2 — Additional file 2. Quality assessment. [file 40834_2021_161_MOESM2_ESM.docx]

**Additional file 2**: Newcastle-Ottawa Quality Assessment Scale for cross sectional studies to assess dual contraceptive utilization among HIV positive women in Ethiopia 2020.

| Authors | Representativeness | Sample size | None-responders | Ascertainment | | comparability | | outcome | Quality score |
| --- | --- | --- | --- | --- | --- | --- | --- | --- | --- |
| Abay et al. (2020) | 1 | 2 | 1 | 1 | 1 | | 1 | | 7 |
| Reta.et al. (2019) | 1 | 2 | 1 | 2 | 1 | | 1 | | 8 |
| Kebede et al. (2015) | 2 | 1 | 1 | 2 | 1 | | 1 | | 8 |
| Egziabher et al. (2015) | 1 | 1 | 1 | 2 | 1 | | 1 | | 8 |
| Tewabe et al. (2018) | 2 | 1 | 1 | 1 | 2 | | 1 | | 8 |
| Asfaw et al. (2014) | 2 | 2 | 1 | 1 | 2 | | 1 | | 9 |
| Demissie et al. (2015) | 1 | 1 | 1 | 2 | 1 | | 1 | | 7 |
| Sufa et al. (2013) | 1 | 2 | 1 | 1 | 2 | | 1 | | 8 |
| Polis et al. (2014) | 2 | 1 | 1 | 1 | 2 | | 1 | | 8 |
| Dereje et al. (2019) | 1 | 2 | 1 | 1 | 2 | | 1 | | 8 |
| Feyissa et al. (2020) | 2 | 1 | 1 | 1 | 2 | | 1 | | 8 |
| Tesfaye et al. (2014) | 1 | 2 | 1 | 1 | 2 | | 1 | | 8 |
| Gebrehiwot et al. (2017) | 2 | 2 | 1 | 1 | 1 | | 1 | | 8 |
| Kalayu (2019) | 2 | 2 | 1 | 1 | 1 | | 1 | | 8 |
| Melaku et al. (2014) | 2 | 2 | 1 | 1 | 1 | | 1 | | 8 |
| Brhane et al. (2013) | 2 | 1 | 1 | 1 | 2 | | 1 | | 8 |
| Assefa et al. (2014) | 2 | 2 | 1 | 1 | 1 | | 1 | | 8 |
| Markos et al. (2019) | 1 | 1 | 1 | 2 | 2 | | 1 | | 8 |
| Meseret et al. (2015) | 1 | 1 | 1 | 1 | 1 | | 1 | | 6 |

Interpretation of the score

Very Good Studies: 9-10 points

Good Studies: 7-8 points

Satisfactory Studies: 5-6 points

Unsatisfactory Studies: 0 to 4 points
